# Supplementary material for: Causal Relationship between Aging and Anorexia Nervosa: A White-Matter-Microstructure-Mediated Mendelian Randomization Analysis
Source: Biomedicines. 2024 Aug 16;12(8):1874. doi: 10.3390/biomedicines12081874 (PMC11351342; doi:10.3390/biomedicines12081874)
Supplement: Supplementary file 1 [file biomedicines-12-01874-s001.zip › Supplementary Figure/Figure S4/Singe SNP plot of MR analysis for TL to ubm-b-1669.pdf]

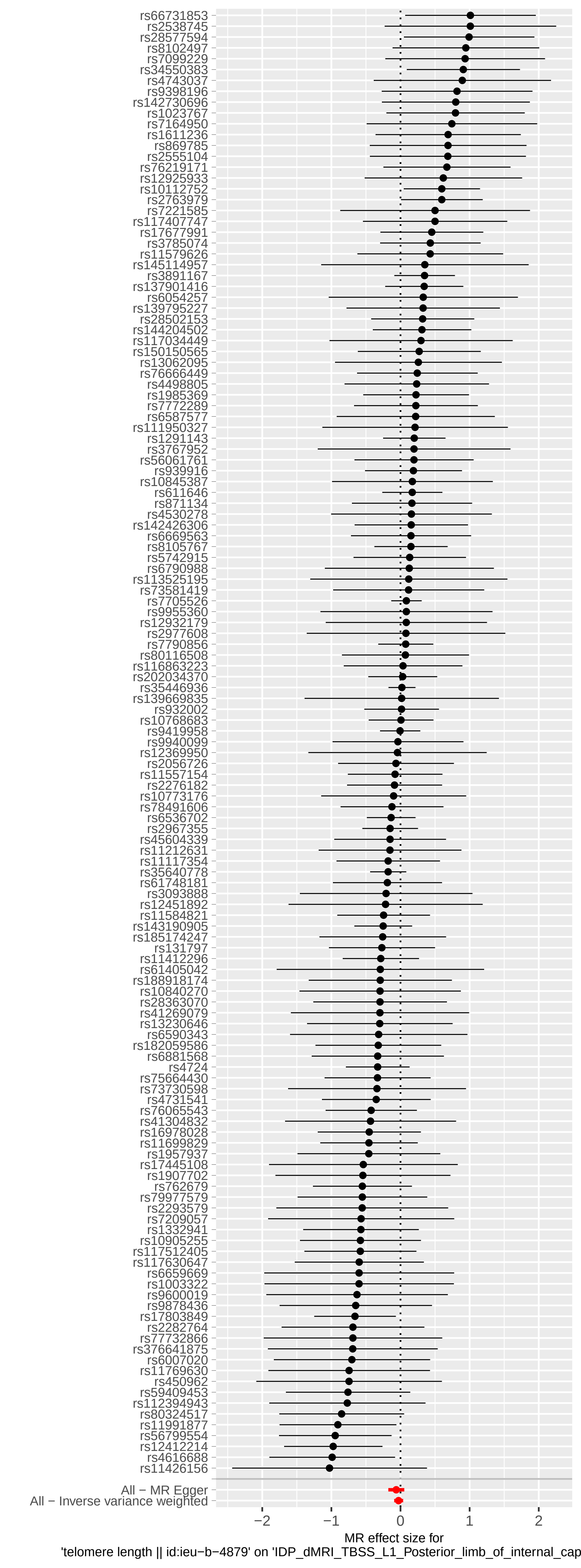

rs66731853
rs2538745
rs28577594
rs8102497
rs7099229
rs34550383
rs4743037
rs9398196
rs142730696
rs1023767
rs7164950
rs1611236
rs869785
rs2555104
rs76219171
rs12925933
rs10112752
rs2763979
rs7221585
rs117407747
rs17677991
rs3785074
rs11579626
rs145114957
rs3891167
rs137901416
rs6054257
rs139795227
rs28502153
rs144204502
rs117034449
rs150150565
rs13062095
rs76666449
rs4498805
rs1985369
rs7772289
rs6587577
rs111950327
rs1291143
rs3767952
rs56061761
rs939916
rs10845387
rs611646
rs871134
rs4530278
rs142426306
rs6669563
rs8105767
rs5742915
rs6790988
rs113525195
rs73581419
rs7705526
rs9955360
rs12932179
rs2977608
rs7790856
rs80116508
rs116863223
rs202034370
rs35446936
rs139669835
rs932002
rs10768683
rs9419958
rs9940099
rs12369950
rs2056726
rs11557154
rs2276182
rs10773176
rs78491606
rs6536702
rs2967355
rs45604339
rs11212631
rs11117354
rs35640778
rs61748181
rs3093888
rs12451892
rs11584821
rs143190905
rs185174247
rs131797
rs11412296
rs61405042
rs188918174
rs10840270
rs28363070
rs41269079
rs13230646
rs6590343
rs182059586
rs6881568
rs4724
rs75664430
rs73730598
rs4731541
rs76065543
rs41304832
rs16978028
rs11699829
rs1957937
rs17445108
rs1907702
rs762679
rs79977579
rs2293579
rs7209057
rs1332941
rs10905255
rs117512405
rs117630647
rs6659669
rs1003322
rs9600019
rs9878436
rs17803849
rs2282764
rs77732866
rs376641875
rs6007020
rs11769630
rs450962
rs59409453
rs112394943
rs80324517
rs11991877
rs56799554
rs12412214
rs4616688
rs11426156
All − MR Egger
All − Inverse variance weighted
−2
−1
0
1
2
MR effect size for
'telomere length || id:ieu−b−4879' on 'IDP_dMRI_TBSS_L1_Posterior_limb_of_internal_cap
